# A brain proteomic investigation of rapamycin effects in the *Tsc1*^+/−^ mouse model

**DOI:** 10.1186/s13229-017-0151-y

**Published:** 2017-08-01

**Authors:** Hendrik Wesseling, Ype Elgersma, Sabine Bahn

**Affiliations:** 1 Department of Chemical Engineering and Biotechnology, University of Cambridge, Tennis Court Road, Cambridge, CB2 1QT UK; 2 Department of Neuroscience, Erasmus Medical Center, Rotterdam, 3000 CA The Netherlands

**Keywords:** Tuberous sclerosis, Rapamycin, Proteomics, SRM, Animal model

## Abstract

**Background:**

Tuberous sclerosis complex (TSC) is a rare monogenic disorder characterized by benign tumors in multiple organs as well as a high prevalence of epilepsy, intellectual disability and autism. TSC is caused by inactivating mutations in the *TSC1* or *TSC2* genes. Heterozygocity induces hyperactivation of mTOR which can be inhibited by mTOR inhibitors, such as rapamycin, which have proven efficacy in the treatment of TSC-associated symptoms. The aim of the present study was (1) to identify molecular changes associated with social and cognitive deficits in the brain tissue of *Tsc1* ^+/−^ mice and (2) to investigate the molecular effects of rapamycin treatment, which has been shown to ameliorate genotype-related behavioural deficits.

**Methods:**

Molecular alterations in the frontal cortex and hippocampus of *Tsc1* ^+/−^ and control mice, with or without rapamycin treatment, were investigated. A quantitative mass spectrometry-based shotgun proteomic approach (LC-MS^E^) was employed as an unbiased method to detect changes in protein levels. Changes identified in the initial profiling stage were validated using selected reaction monitoring (SRM). Protein Set Enrichment Analysis was employed to identify dysregulated pathways.

**Results:**

LC-MS^E^ analysis of *Tsc1* ^+/−^ mice and controls (*n* = 30) identified 51 proteins changed in frontal cortex and 108 in the hippocampus. Bioinformatic analysis combined with targeted proteomic validation revealed several dysregulated molecular pathways. Using targeted assays, proteomic alterations in the hippocampus validated the pathways “myelination”, “dendrite,” and “oxidative stress”, an upregulation of ribosomal proteins and the mTOR kinase. LC-MS^E^ analysis was also employed on *Tsc1* ^+/−^ and wildtype mice (*n* = 34) treated with rapamycin or vehicle. Rapamycin treatment exerted a stronger proteomic effect in *Tsc1* ^*+/−*^ mice with significant changes (mainly decreased expression) in 231 and 106 proteins, respectively. The cellular pathways “oxidative stress” and “apoptosis” were found to be affected in *Tsc1* ^+/−^ mice and the cellular compartments “myelin sheet” and “neurofilaments” were affected by rapamycin treatment. Thirty-three proteins which were altered in *Tsc1* ^+/−^ mice were normalized following rapamycin treatment, amongst them oxidative stress related proteins, myelin-specific and ribosomal proteins.

**Conclusions:**

Molecular changes in the *Tsc1* ^+/−^ mouse brain were more prominent in the hippocampus compared to the frontal cortex. Pathways linked to myelination and oxidative stress response were prominently affected and, at least in part, normalized following rapamycin treatment. The results could aid in the identification of novel drug targets for the treatment of cognitive, social and psychiatric symptoms in autism spectrum disorders. Similar pathways have also been implicated in other psychiatric and neurodegenerative disorders and could imply similar disease processes. Thus, the potential efficacy of mTOR inhibitors warrants further investigation not only for autism spectrum disorders but also for other neuropsychiatric and neurodegenerative diseases.

**Electronic supplementary material:**

The online version of this article (doi:10.1186/s13229-017-0151-y) contains supplementary material, which is available to authorized users.

## Background

Tuberous sclerosis complex (TSC) is a rare multi-system monogenic hamartomatous disorder, which is caused by mutations inactivating the *TSC1* (hamartin) or *TSC2* (tuberin) genes. TSC is characterized by benign tumors in multiple organs, including the brain, kidneys, heart and eyes [1]. Over 90% of TSC patients develop epilepsy, and around 50% present with neuropsychiatric problems, such as intellectual disability (50%) [2, 3], autism spectrum disorder (ASD) (17–68%), schizophrenia (10–30%) and anxiety disorders (40%) [4], which account for most of the mortality and morbidity [5].

At the molecular level, both *Tsc1* and *Tsc2* protein products form hetero-dimers which inhibit the GTP-binding protein RHEB (Ras homolog enriched in the brain). Consequently, mutations within either *Tsc1* or *Tsc2* lead to increased levels of activated RHEB [6], which causes hyperactivation of mammalian target of rapamycin (mTOR) signaling, a constitutive phosphorylation of eukaryotic translation initiation factor 4E-binding protein 1 (4E-BP1) and activation of ribosomal protein S6 through S6K1 phosphorylation [7, 8]. The net effect is enhanced protein translation, cell proliferation and growth [9]. Notably, increased mTOR signaling and subsequent changes in global protein synthesis are shared molecular mechanisms of several rare neurodevelopmental disorders with an increased prevalence of ASD, such as fragile X syndrome (FXS) [10].

The hyperactivation of mTOR induced by *Tsc1* and *Tsc2* heterozygosity can be inhibited by mTOR inhibitors, such as the macrolide rapamycin. Rapamycin is an immunosuppressant, which is widely prescribed to prevent rejection in organ transplantation and exerts anti-tumor properties [11–13]. Rapamycin binds FK-binding protein 12 (FKBP12), and as a complex, rapamycin-FKBP12 directly binds to the mTOR complex 1 (mTORC1), thus reducing phosphorylation of downstream mTOR targets [14, 15]. Rapamycin and other mTOR inhibitors have been shown to be efficacious in the treatment of several TSC-associated tumors as well as seizures [16–19] and may ameliorate the symptoms of neurodevelopmental disorders in adults [20, 21]. In TSC mouse models, rapamycin limits tumor growth [22, 23], reduces neuropathology and ameliorates epileptic seizures as well as learning deficits [24–26]. It was recently reported that rapamycin normalizes social interaction deficits relevant to core disabilities associated with ASD in both *Tsc1* ^+/−^ and *Tsc2* ^+/−^ mice [27].

Here, we investigated the *Tsc1* ^+/−^ mouse model, which exhibits haploinsufficiency for the *Tsc1* gene, in an attempt to identify molecular changes associated with the neuropsychiatric phenotype of TSC patients [5]. In this mouse model, the typical human cerebral pathology of spontaneous seizures, cerebral lesions and giant dysmorphic cells could not been detected using immuno-cytochemistry and high resolution magnetic resonance imaging, respectively [28]. Furthermore, spine number and dendritic branching are normal [28]. However, the *Tsc1* ^*+/−*^ mouse shows prominent behavioural deficits which mimic core symptoms of ASD and other neuropsychiatric disorders [28]. *Tsc1* ^+/−^ mice show hippocampal learning deficits using the Morris water maze test and contextual fear conditioning, as well as social deficits indicated by reduced social interaction and nest building [28]. Consequently, the *Tsc1* ^+/−^ mouse is a suitable model to investigate aspects of the molecular pathology associated with neuropsychiatric spectrum disorders, especially in relation to ASD and intellectual disability. In this study, we attempted to identify changes in molecular pathways in the frontal cortex and hippocampus of the *Tsc1* ^+/−^ mouse model using a mass spectrometry-based proteomics approach. We also investigated protein changes associated with rapamycin treatment. Findings from this study could aid in the identification of novel drug targets for the treatment of cognitive, social and psychiatric symptoms in ASD.

## Methods

A more detailed description of the materials and methods used in this study can be found in the supplementary methods section (Additional file 1).

### Animals

*Tsc1* ^+/−^ mice were generated by replacing exons 6 through to 8 of the *Tsc1 gene* with a selection cassette, as described previously [29]. This leads to the generation of *Tsc1* null embryos which express *Tsc1* transcripts in which exon 5 and 9 are fused, leading to a premature TGA stop codon. Consequently, any protein translated from this allele lacks all of the known functional domains of hamartin including the putative Rho activation domain. The *Tsc1* ^*+/−*^ mutant mouse was crossed six times into the C57BL/6J OlaHsD background and then at least three times into the C57BL/6N/HsD background. The offspring consisted of *Tsc1* ^+/−^ mice and wildtype littermates. Mice were genotyped when they were about 7 days old. They were housed in groups and kept on a 12-h light/dark cycle, with food and water available ad libitum. Mice were culled when they were 6–8 weeks old and genotype groups were sex- and age-matched for the experiments for consistency with the published behavioural data [28]. Mouse genotypes were blinded using codes all the way through to the sample preparation stage. The codes were un-blinded for the mass spectrometry analysis since samples had to be distributed evenly to avoid run time biases. All animal experiments were approved by the Dutch Ethical Committee or in accordance with Institutional Animal Care and Use Committee guidelines.

### Rapamycin treatment

Mice were injected intraperitoneally with 5 mg/kg rapamycin or vehicle for 5 days and culled 24 h after the last injection [27]. Rapamycin was dissolved in 5% dimethyl sulfoxide diluted with saline to 5 ml/kg. Mice were 5–7 weeks old at the time of injection.

### Proteomic sample preparation

Sample preparation was carried out as described previously [30–32]. Based on the lysates, two randomized, blinded, independent sample preparations were prepared for liquid chromatography mass spectrometry (in expression mode; LC-MS^E^) and selected reaction monitoring mass spectrometry to avoid bias in sample preparations.

### Label-free LC-MS^E^ proteomic profiling of brain tissue

Brain tissue analysis and data processing were performed as described previously [31, 33, 34]. The Swiss-Prot human reference proteome (Uniprot release March 2013, 20,252 entries) was used for protein identification searches. Protein abundance changes for the comparisons between *Tsc1* ^*+/−*^ and wildtype were determined by the MSstats package [35] based on mixed-effect models using the peptide intensities, following log_2_ transformation and exclusion of intensity values deviating by more than 3 standard deviations from the mean of each group.

### Protein set enrichment analysis

Significantly changed proteins were partitioned into three bins, according to their ratio: proteins decreased in abundance (ratio < 1.0), proteins increased in abundance (ratio > 1.0) and a bin to identify general disturbed pathways which included all proteins with increased and decreased abundance (ratio > 1 and <1). The *R* package database org.mouse.eg.db version 2.8.0 was used for gene ontology (GO) term annotation based on entrez gene identifiers and GO-term enrichment analysis was performed using *GOstats*.

### Label-based SRM mass spectrometry

Abundance alterations of a panel of 43 candidate proteins previously implicated in the *Tsc1* ^*+/−*^ mouse pathology were measured using a targeted SRM mass spectrometry approach as described previously [32, 36] following the guidelines of Lange et al. [37]. SRMstats was used at default settings [37]. The final transitions, collision energy and retention time windows used for each peptide can be requested.

## Results

### Label-free LC-MS^E^ proteomic profiling of *Tsc1*^*+/−*^ mouse brains

We investigated protein abundance changes in the frontal cortex and hippocampus of the *Tsc1* ^+/−^ mouse. LC-MS^E^ analysis resulted in the identification of 522 proteins (7071 peptides) in the frontal cortex and 463 proteins (5149 peptides) in the hippocampus. Of these, the levels of 51 proteins were altered in the frontal cortex (FDR-adjusted *p < 0.05) and 108 in the hippocampus (FDR-adjusted *p < 0.05). In the frontal cortex, 17 of the changed proteins were altered by more than 10%, as were 49 of the 108 changed hippocampal proteins (Additional file 2). In the case of the frontal cortex, this included adenylyl cyclase-associated protein 2 (CAP2, ratio = 0.89, FDR-adjusted *p = 0.013), elongation factor 1–α2 (EIF1A2, ratio = 0.97, FDR-adjusted *p = 0.03), eukaryotic translation initiation factor 3 subunit L (eIF3l, ratio = 1.17, FDR-adjusted *p = 0.03) and elongation factor 2 (Eef2, ratio = 0.95, FDR-adjusted *p = 0.05), which are all regulators of translation. Copine 6 (ratio = 1.1, FDR-adjusted *p* = 0.0097) and copine 8 (ratio = 0.8, FDR-adjusted *p* = 3.9 × 10^−6^), which are associated with synaptic plasticity, were changed in the hippocampus. Nine proteins (NCDN⬇⬇, MAP2⬆⬆, SUCB1⬇⬇, MYPR⬆⬇, NDUS7⬇⬇, DPYL2⬇⬇, AT1A2⬇⬆, CRYM⬇⬆, ARP3⬆⬇) were found to be changed in both frontal cortex (first arrow) and hippocampal tissue (second arrow) (Additional file 2).

Gene set enrichment analysis was employed to investigate if the altered 108 and 51 proteins were enriched in biological pathways and cellular compartments. Based on GO enrichment analysis, proteins responsible for the biological pathways “reproductive behaviour” (*p* = 0.008), “neurological system process” (*p* = 0.010) and “visual learning” (*p* = 0.028) were altered in the frontal cortex of the *Tsc1* ^+/−^ mouse. In the hippocampus, the proteins were related to the biological pathways “ribonucleotide energy metabolism” (*p* = 0.0097), “protein polymerisation” (*p* = 0.005) and “oxidative stress” (*p* = 0.009). One pathway, “visual learning”, was identified in both the frontal cortex and hippocampus proteomic analyses. Cellular compartment GO association enrichment revealed that the altered proteins were associated with “myelination” and “dendrite” in the frontal cortex and “myelin sheet” and “endoplasmic reticulum-Golgi intermediate compartment” in the hippocampus.

### Selected reaction monitoring (SRM) validation of *Tsc1*^+/−^ brain proteomic alterations

For orthogonal proteomic validation of the proteomics results, we employed a targeted label-based LC-SRM approach to specifically quantify the levels of 43 candidate proteins derived from LC-MS^E^ profiling, subsequent pathway analysis, literature findings and already established in-house assays. Label-based SRM assays are highly specific and sensitive and outperform immunoblotting analyses for validation [38–41]. This analysis showed that the levels of 5 and 20 of the targeted proteins were significantly changed in the frontal cortex and hippocampus, respectively (*p* < 0.05, Table 1). Specifically, two proteins (MYPR, PURA) out of five LC-MS^E^ candidates were validated in the frontal cortex and six (NSF, MYPR, MBP, CPNE6, SODC, MARCS) out of ten LC-MS^E^-derived protein candidates were validated in the hippocampus (Table 1). Using targeted SRM-assays, we confirmed the more prominent proteomic alterations in the hippocampus and further validated GO-terms “myelination”, “dendrite” and “oxidative stress” through confirmation of changes in MYPR, MBP, TSN2 (all myelin specific proteins), MAP2 (dendritic marker) and SODC (oxidative stress marker). We further detected an upregulation in ribosomal proteins and mTOR kinase in the *Tsc1* ^*+/−*^ mice (Table 1).

**Table 1:**
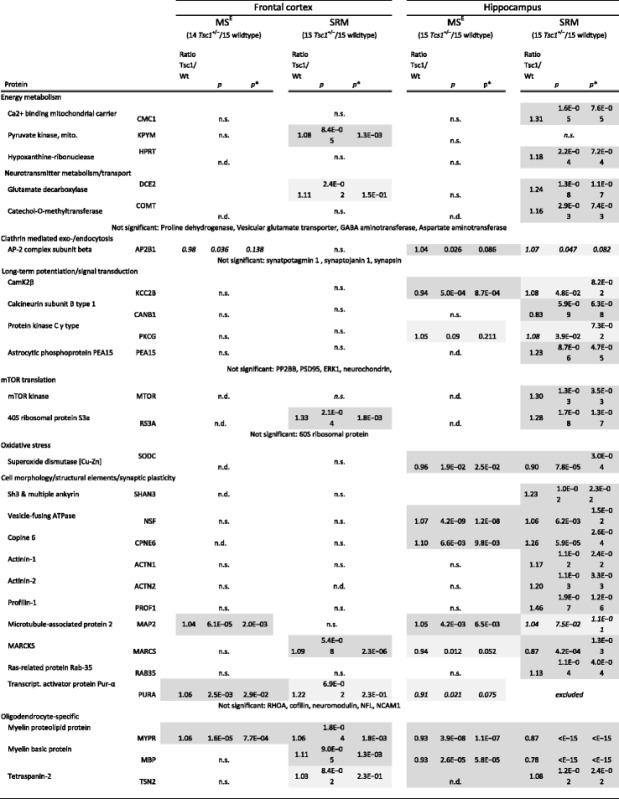
Significantly changed proteins identified by label-based LC-SRM in the frontal cortex and hippocampus of *Tsc1* ^+/−^ mice compared to wildtype mice

The first stage of the analysis consisted of a global profiling approach, followed by validation with a specific and sensitive label-based assay panel. *p* values were determined using SRMstats (linear model with fixed subject effects) and corrected (p*) to control for multiple hypothesis testing (Benjamini-Hochberg) [90]. For reasons of clarity, only ratios and significance levels of significantly changing proteins are shown. For full information, see Additional file 3. *n.s.* not significant, *n.d.* not detected. Validated findings are in gray shading

### Label-free LC-MS^E^ proteomic profiling of the *Tsc1*^+/−^ hippocampus following rapamycin treatment

**Fig. 1:**
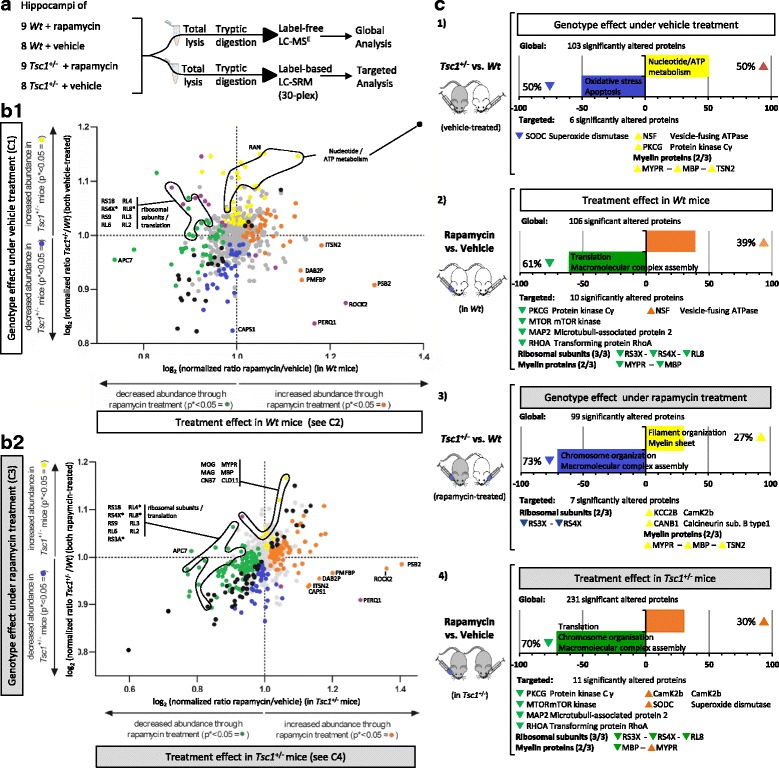
Label-free LC-MSE and Label-based SRM analysis of Tsc1+/− and wildtype mice under rapamycin and vehicle treatment identifies distinct proteomic changes. **a**) Flow chart of the experimental design. **b**) Significant protein changes in the hippocampus of rapamycin-treated WT and *Tsc1* ^+/−^ mice identified by label-free LC-MS^E^. *Orange dots* refer to proteins that are increased in abundance, and *green dots* represent downregulated proteins following rapamycin treatment. **c** *2* and *4* (see below) refer to significantly changed proteins following rapamycin treatment in wildtype (“treatment effect in Wt mice”) or Tsc1^+/−^ mice (“treatment effect in *Tsc1* ^+/−^ mice”). Protein enrichment analysis was performed on the identified proteins. *Yellow* and *blue dots* represent the TSC genotype effect following rapamycin or vehicle treatments (**b** *1* and *2*) and are linked to **c** *1* and *3*. Proteins changing due to a combination of TSC genotype and rapamycin treatment are labeled *black* or *purple*, respectively. **b** *1* Rapamycin induced changes in wildtype mice as compared to *Tsc1* ^+/−^ mice following vehicle treatment. **b** *2* Rapamycin induced changes in *Tsc1* ^+/−^ mice compared to *Tsc1* ^+/−^ alterations following rapamycin treatment. **c**) Bar plots of genotype and treatment effects identified through global protein profiling and significantly changed proteins identified in the targeted SRM analysis. Number of significant proteins, percentage of up- and downregulated proteins and enriched pathways linked to the up- and downregulated proteins are displayed

To investigate the effects of rapamycin on the brain proteome, label-free LC-MS^E^ analysis was employed on *Tsc1* ^+/−^ and wildtype mice treated with rapamycin or vehicle (Fig. 1a). Only the hippocampus was studied in this case as this brain region was more affected with regard to significantly changed proteins (Table 1). The hippocampus plays not only an important role in cognition, but hippocampal dysfunction has also been linked to a wide range of neuropsychiatric symptoms [42, 43]. Deficits in consolidating short- and long-term memory and spatial navigation have been shown to be impaired in *Tsc1* ^*+/−*^ and Tsc2^+/−^ mice and were reversed by rapamycin treatment in Tsc2^+/−^ mice [24]. LC-MS^E^ analysis led to the identification of 8648 total peptides which translated to 597 proteins, which were detected across all samples. Interestingly, rapamycin treatment exerted a stronger proteomic effect in *Tsc1* ^*+/−*^ compared to wildtype mice (Fig. 1c (2 and 4)) with significant changes in 231 and 106 proteins, respectively. An overall decrease in protein levels was found in both *Tsc1* ^*+/−*^ and wildtype mice.

Next, proteins were tested which were affected in all four comparisons. This showed that 9 proteins were changed in common (FRM4A, PEA15, PERQ1, MAP2, BASP, CLD11, ALBU, TCAL3, CLH) and that the levels of 54 proteins were affected by rapamycin treatment in both wildtype and *Tsc1* ^+/−^ mice; of these proteins, 52 corresponded in their fold change direction (37 of the 52 proteins were decreased in abundance and 15 increased, respectively). Pathway analysis linked the 52 overlapping proteins to the biological process of “translation” (*p* = 0.00082), “macromolecule biosynthetic process” (*p* = 0.005) and “gene expression” (*p* = 0.014). Using KEGG (Kyoto Encyclopedia of Genes and Genomes) annotation, “ribosome” was the most significant pathway (*p* = 1.7 × 10^−7^) in the enrichment analysis.

We further employed enrichment analysis for the genotype comparisons and the treatment comparisons (Fig. 1c (1–4)). This associated the biological pathways “oxidative stress” and “apoptosis” with the significantly changed proteins identified in the *Tsc1* ^+/−^ vs Wt comparison (Fig. 1c (1)). Furthermore, cellular compartments of myelin sheet and neurofilaments were affected by rapamycin treatment in both *Tsc1* ^+/−^ and Wt mice (Fig. 1c (2 and 4)). Proteins with decreased levels due to rapamycin treatment mostly related to the biological pathways “translation”, “macromolecular complex assembly” and “chromosome organization” (Fig. 1c (2 and 4)). Downregulation of the pathway “chromosome organization” was specifically observed in *Tsc1* ^+/−^ mice following rapamycin treatment (Fig. 1c (4)).

**Fig. 2:**
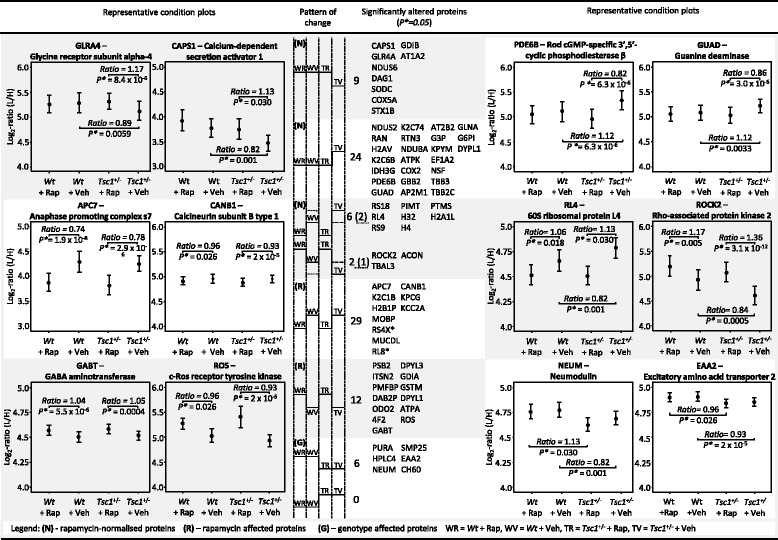
Multivariate analysis of LC-MS^E^ estimates as shown by condition plots illustrating the differences between the WT and *Tsc1* ^+/−^ mice with and without rapamycin treatment. *X*-axis is condition and *y*-axis is log ratio of endogenous (*L* light) over reference (*H* heavy) peptides. *Dots* represent the mean of the log_2_ ratio for each condition, and *error bars* indicate the confidence intervals with 0.95 significance. The interval is not related to the model based analysis. Significant changes as measured by LC-MS^E^ are indicated below each protein. Illustrated are examples of proteins which are affected by rapamycin treatment and which are normalized by rapamycin treatment. Corrected *p* values (p*) were determined by post hoc correction after Benjamini-Hochberg [91]. CR = *Wt* + rapamycin, CS = *Wt* + vehicle, TR = *Tsc1* ^+/−^ + rapamycin, TS = *Tsc1* ^+/−^ − rapamycin

Importantly, 41 proteins which were altered in vehicle-treated *Tsc1* ^+/−^ mice were normalized following rapamycin treatment. These proteins include a set of proteins where rapamycin treatment normalizes the genotype-induced protein alterations to wildtype levels (33 proteins) and a set of proteins where rapamycin normalizes the genotype-effect below or above baseline levels (8 proteins). The former include the Glycine receptor subunit alpha-4 (GLRA4), the Calcium-dependent secretion activator 1 (CAPS1), Rod cGMP-specific 3′,5′ cyclic phosphodiesterase beta (PDE6B) and Guanine deaminase (GUAD) (Fig. 2 (N)); the latter include Rho-associated protein kinase 2 (ROCK2) and ribosomal proteins (RS18, RL4, RS9). All ribosomal proteins affected by rapamycin treatment were decreased in their abundance levels. Furthermore, proteins were identified that are affected by rapamycin treatment in both wildtype and mutant mice, although there was no difference in their abundance levels between vehicle-treated mutant and wildtype mice (Fig. 2 (R)). This set was comprised of 41 proteins. Amongst them are the anaphase promoting complex s7 (APC7), calcineurin subunit B type 1 (CANB1) and the GABA aminotransferase (GABT). Finally, the levels of six proteins were found to be altered between mutant and wildtype but did not change following rapamycin treatment. Neuromodulin (NEUM), the excitatory amino acid transporter 2 (EAAT2) and SMP25 are examples.

### SRM validation of rapamycin treatment effects in the *Tsc1*^+/−^ hippocampus

The next phase of the study involved a targeted proteomic approach to validate the findings of the rapamycin study (see Fig. 1c (1–4) [targeted]). This focused on myelination deficits, alterations in the translational machinery and proteins found to be altered in the label-free LC-MS^E^ discovery study of the *Tsc*1^+/−^ mouse (SODC, NSF, MAP2, PKCG). The rapamycin-target mTOR was included as a positive control.

**Fig. 3:**
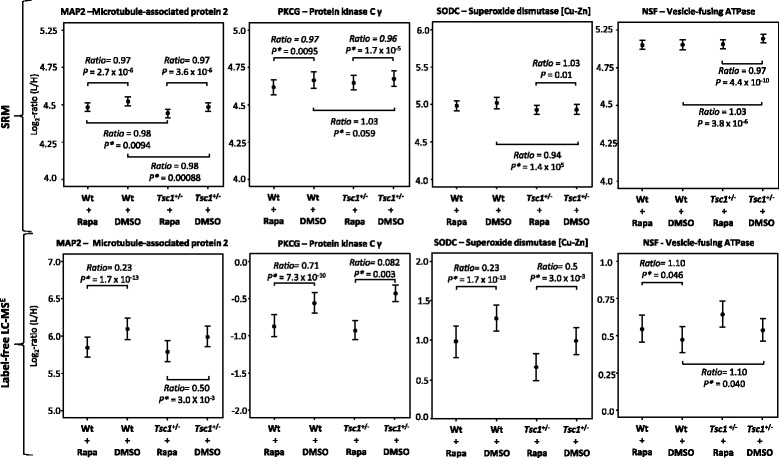
Multivariate analysis of SRM estimates as shown by condition plots illustrating the differences between Wt and *Tsc1* ^+/−^ mice with and without rapamycin treatment. *X*-axis represents the condition and the *y*-axis the log ratio of endogenous (*L* light) over reference (*H* heavy) peptides. *Dots* represent the mean of log_2_ ratio for each condition, and *error bars* indicate the confidence intervals with 0.95 significance. The interval is not related to the model based analysis. Significant changes as measured by LC-MS^E^ are indicated below each protein. SRM was able to validate protein abundance changes identified by label-free LC-MS^E^. Corrected *p* values (p*) were determined by post hoc correction after Benjamini-Hochberg [91]

This analysis resulted in validation of increased myelin-associated proteins in *Tsc1* ^+/−^ compared to wildtype mice (Fig. 1c (1 and 3)). Furthermore, the decrease in oxidative stress-related proteins in the *Tsc1* ^+/−^ mouse was validated by confirming decreased levels of SODC (Fig. 1c (1)). Additionally, altered levels of NSF and PKCG were also validated (Fig. 1c [targeted] and Fig. 3).

In the case of rapamycin treatment effects, decreased levels of proteins involved in translation could be validated. Specifically, decreased levels of all three tested ribosomal subunits as well as the mTOR-kinase were identified. Moreover, the analysis showed that rapamycin normalized the protein levels of SODC, NSF and PKCG in *Tsc1* ^+/−^ mice (Fig. 1c [targeted]).

## Discussion

The pathogenesis of psychiatric disorders such as ASD remains elusive, and there is accumulating evidence that several neuronal circuits and pathways are affected. This is especially true for the social, cognitive and neuropsychiatric symptoms associated with these disorders. In an attempt to gain further insight into these pathways, this study combines unbiased and targeted proteomic approaches to investigate the hippocampus and frontal cortex of a mouse model of TSC, which is one of the most frequent causes of syndromic ASD [44]. The investigated *Tsc1* ^+/−^ mouse model exhibits social and cognitive deficits, which are core behavioural symptoms of ASD in humans [45] and other relevant rodent models [46], without any obvious brain pathology (such as tumors or epilepsy). This makes the *Tsc1* ^+/−^ mouse an excellent model of pharmacologically treatable ASD. A previous study has demonstrated the effectiveness of rapamycin to normalize reciprocal social interactions in this model [27]. The aim of the present study was to investigate proteins and pathways affected by rapamycin treatment, which could support drug discovery efforts and in turn the development of improved treatments for TSC, ASD and possibly other neuropsychiatric disorders.

Proteomic profiling of the frontal cortex and hippocampus brain tissue in this study identified and quantified a large number of significantly changed proteins in the *Tsc1* ^+/−^ mouse model. Differentially expressed proteins and altered molecular pathways were identified and selected candidate proteins were validated using SRM as a highly quantitative method. In a second stage, proteomic analysis of rapamycin treatment effects were investigated to identify down-stream effects of mTOR-pathway inhibition in the hope to gain new insights into the molecular underpinnings of social impairments in ASD and other psychiatric disorders.

We were able to show that myelin proteins and the translational machinery, specifically several ribosomal subunits, were significantly altered in *Tsc1* ^+/−^ mice treated with rapamycin. Our findings of lower ribosomal subunit abundances are consistent with a rapamycin-induced downregulation of ribosomal biogenesis [47]. Regarding the effects on myelination, previous research has linked the mTOR pathway to oligodendrocyte differentiation and axonogenesis [48]. Oligodendrocytes produce myelin, and this is specifically regulated at the late progenitor to immature oligodendrocyte transition stage (as shown by changes in expression of the myelin marker proteins MYPR and MBP). We identified an increase in myelin proteins in the *Tsc1* ^+/−^ mouse, but not in Wt mice. A recent study has shown that ablation of TSC1 is associated with oligodendrocyte-specific over-activation and subsequent hypomyelination [49]. An increase in myelin proteins in the *Tsc1* ^+/−^ mouse brain may be due to the globally enhanced (Table 1) protein translation and cell proliferation in the context of mTOR hyperactivation. An increase in cell growth and proliferation could interfere with oligodendrocyte maturation and thus result in incomplete myelination as seen in some demyelinating diseases, such as multiple sclerosis [50].

MYPR and MBP as well as TSN-2 were amongst the altered myelin proteins. These proteins play an important role in oligodendrocyte differentiation during development. Furthermore, in vitro studies have shown that MBP mRNA and protein expression are significantly decreased by mTOR inhibition [48, 51]. Inhibiting mTOR in oligodendrocyte precursor cell/dorsal root ganglion co-cultures potently abrogated oligodendrocyte differentiation and reduced numbers of myelin segments. Disorganized and structurally compromised axons with poor myelination have already been found in TSC patients, and this may at least to some extent explain the behavioural and cognitive deficits associated with the disorder [52]. Impaired adult myelination has been shown in the prefrontal cortex of socially isolated mice [53]. Importantly, changes in oligodendrocyte function and myelination abnormalities are amongst the most consistent hallmarks of psychiatric pathology in post-mortem brain studies. Changes were reported for schizophrenia, bipolar disorder, depression and ASD [36, 54–58]. In wildtype mice, rapamycin treatment led to a reduction of myelin and myelin protein expression [59, 60]. This is consistent with our findings, where rapamycin affects both wildtype (reduced) and mutant myelin (increased) protein expression.

Interestingly, several proteins that we found altered in the *Tsc1* ^+/−^ mouse model were reversed by rapamycin treatment. One of these proteins, a glycine receptor subunit, which abundance was decreased in the mutant and normalized by rapamycin treatment, could be a potential drug target for novel treatments of ASD and schizophrenia-spectrum disorders. The glycine receptor co-localizes with GABA_A_ receptors on hippocampal neurons [61]. A microdeletion at Xq22.2 implicates GLRA4 to be involved in intellectual disability and behavioural problems [62]. In a case report, glycine receptor antibodies could be detected in a patient with treatment-resistant focal epilepsy, tantrums, clumsiness and impaired speech [63] and in patients with progressive encephalomyelitis with rigidity and myoclonus stiff person syndrome [64]. Treatments targeting the glycine transporter are under investigation as novel treatment approaches for schizophrenia [65].

Other altered proteins, which are normalized by rapamycin treatment, included the calcium-dependent secretion activator 1 (CAPS1) (decreased in *Tsc1* ^+/−^ and normalized by rapamycin), two guanine metabolism associated proteins (guanine deaminase and PDE6B; both increased in *Tsc1* ^+/−^ and normalized by rapamycin) and the vesicle-fusing ATPase NSF (increased in *Tsc1* ^+/−^, normalized by rapamycin), a molecular component of the exocytosis machinery [66], which is required for membrane fusion [67] and regulates the disassembly of SNARE complexes on early endosomes [68]. The NSF gene has also been linked with cocaine dependence [69] and schizophrenia [36, 70]. Direct interactions with cell surface receptors such as AMPA receptors [71, 72], β2-adrenergic receptors [73], dopaminergic receptors [74] and the adrenomedulin receptor [75] have been reported. Interestingly, a coordinated action of NSF and PKC regulates GABA_B_ receptor signaling efficiency [76]. PKCG was found to be strongly downregulated by rapamycin treatment in this study and is known to be involved in the regulation of the neuronal receptors GLUR4 and NMDAR1 [77]. It binds and phosphorylates the GLUR4 glutamate receptor and regulates its function by increasing membrane-associated GRIA4 expression [78]. Several preclinical and clinical trials have investigated mGLUR antagonists for the treatment of social deficits in ASD [78, 79] and ASD associated with FXS [80, 81] and PKCG inhibitors could represent a novel treatment strategy to ameliorate cognitive and social deficits. Notably, Ketamine, which is thought to exert antidepressant action through modulation of mTOR pathway activity [82], potentiates persistent learning and memory impairment through the PKCG-ERK signaling pathway [83].

Another protein strongly downregulated following rapamycin treatment is the anaphase promoting complex S7 (APC7), which is a cell cycle-regulated E3 ubiquitin ligase controlling progression through mitosis and the G1 phase of the cell cycle. The control of APC7 through rapamycin might be a major breakpoint in cell proliferation. Rapamycin has already been shown to also downregulate the expression of the APC/C inhibitor Emi1 [84].

Copine 6, which we found upregulated in the hippocampus of *Tsc1* ^+/−^ compared to wildtype mice, is a calcium-dependent regulator of the actin cytoskeleton in neuronal spines and negatively regulates spine maturation during neuronal development [85]. Changes in copine 6 expression may be involved in neurodevelopmental disorders, as deformed dendritic spines and changes in spine density are hallmarks of many neurodevelopmental conditions, such as Down’s syndrome [86, 87] and FXS [88]. Interestingly, hippocampi from patients suffering from uncontrolled epileptic seizures, typically a problem in tuberous sclerosis patients, exhibit a decrease in spine density [89]. We also found that MAP2, a dendritic spine marker, was increased by *Tsc1* heterozygocity and decreased by rapamycin treatment.

Interestingly, over twice as many significantly changed proteins were identified in the *Tsc1* ^+/−^ hippocampus as compared to control animals following rapamycin treatment (Fig. 1c; 231 vs. 106 changed proteins, respectively). TSC1 mutations are linked to numerous changes in biochemical processes, including cell cycle regulation, translational control and metabolism which are linked to mTOR pathway hyperactivation. It can be speculated that rapamycin-related inhibition of the mTORC1 complex results in TSC genotype-dependent adaptations in a wide range of molecular pathways. These adaptations could be indirectly involved in the therapeutic effect of rapamycin. A further explanation for the enhanced rapamycin treatment effect in the Tsc1^+−/^ mice is selective vulnerability. Mutant mice might be more susceptible to the treatment as mTOR hyperactivation modulated similar downstream molecular pathways during neurodevelopment as are affected by the rapamycin-induced mTOR hypoactivation.

## Conclusions

Taken together, the results from this comprehensive study represent the first proteomic characterization of the *Tsc1* ^+/−^ mouse model to date. The findings yield novel insights into the molecular *Tsc1* ^+/−^ mouse pathology as well as the molecular effects of rapamycin treatment, which is an effective treatment for several clinical symptoms of the tuberous sclerosis complex. Furthermore, the mTOR pathway, which is modulated by rapamycin treatment, is a novel drug target for the treatment of ASD, schizophrenia and affective disorders. We hope that the findings from this study will provide evidence and support for future clinical trials in the field of neuropsychiatric disorders.

## Additional files

Additional file 1: Supplementary methods. Detailed information of the experimental methods. (DOCX 31 kb)

Additional file 2: Significantly altered proteins identified by label-free LC-MS^E^ analysis in the frontal cortex and hippocampus of the *Tsc1* ^+/−^ mice compared to *Wt* mice. Overlapping proteins between hippocampus and frontal cortex are bold. (XLSX 23 kb)

Additional file 3: Full information for significantly changed proteins identified by label-based LC-SRM in the frontal cortex and hippocampus of *Tsc1* ^+/−^ mice compared to wildtype mice. (DOCX 31 kb)

## Abbreviations

ASD: Autism spectrum disorder
LC: Liquid chromatography
MBP: Myelin basic protein
mTOR: Mammalian target of rapamycin
MYPR: Myelin proteolipid protein
NSF: Vesicle-fusing ATPase
PKCG: Protein kinase C gamma
SODC: Superoxide dismutase C
SRM: Selected reaction monitoring
TSC: Tuberous sclerosis complex
TSC1: Harmartin
TSC2: Tuberin
TSN-2: Tetraspanin 2
Wt: Wildtype
