# Supplementary material for: A brain proteomic investigation of rapamycin effects in the Tsc1+/− mouse model
Source: Mol Autism. 2017 Aug 1;8:41. doi: 10.1186/s13229-017-0151-y (PMC5540199; doi:10.1186/s13229-017-0151-y)
Supplement: Supplementary file 1 — Supplementary methods. Detailed information of the experimental methods. (DOCX 31 kb) [file 13229_2017_151_MOESM1_ESM.docx]

**Sample preparation**

Lysis buffer was added to frozen tissue samples containing 7M urea, 2M thiourea, 4 % chaps, 2 % ASB14, 70mM DTT and protease inhibitor at a 5:1 (v/w) ratio.(Martins-de-Souza *et al*, 2007). Samples were sonicated (10 sec, 2 cycles) and vortexed at 4°C for 30 min. Samples were then centrifuged at 17000 g at 4°C. Protein concentrations of the lysates were determined using a Bradford assay (Bio-Rad; Hemel Hempstead, U.K). Approximately 100 μg was precipitated using acetone. Post dissolving the precipitate in 50 mM ammonium bicarbonate, reduction of sulfhydryl groups were performed with 5 mM DTT at 60°C for 30 min and alkylation was carried out using 10 mM iodacetamide in the dark at 37°C for 30 min and subsequently digested using trypsin at a 1:50 (w/v) ratio for 17 h at 37°C. Reactions were stopped by the addition of 8.8 M HCl in a 1:60 (w/w) ratio. Quality control (QC) samples were prepared to monitor machine and preparation performance and randomly distributed across the whole sample and mass spectrometrical analysis.

**Label-free LC−MS^E^ analysis**

Brain tissue samples were analysed individually in technical duplicates. Splitless nano-ultra-performance liquid chromatography (UPLC) (10 kpsi nanoAcquity; Waters Corporation, Milford, MA), was coupled online through a New Objective nanoESI emitter (7 cm length, 10-mm tip; New Objective, Woburn, MA) to a Waters Q-TOF Premier mass spectrometer. Data were acquired in expression mode (MS^E^) and the total continuous run time was 8 days. The procedure, quality assessment and data processing were performed as described previously.(Ernst *et al*, 2012) LC-MS^E^ data were processed by the ProteinLynx Global Server (PLGS v.2.4 Waters, Milford, MA, USA) for ion detection, extraction and identification using an ion accounting algorithm.(Bateman *et al*, 2002) The Swiss-Prot rodent reference proteome database (reviewed entries, 2013) was used for protein identification searches. To control the false discovery rate (FDR), data were searched against a decoy database, which was the randomised version of the database mentioned above to conserve amino acid frequencies. The FDR was set at the default maximum rate of 4%, as applied before.(Krishnamurthy *et al*, 2011; Lu *et al*, 2007; Ralhan *et al*, 2011; Yang *et al*, 2011c) The search parameters were (i) enzyme = trypsin, (ii) fixed modification = carbamidomethylation of cysteines, (iii) variable modifications = oxidation of methionine and phosphorylation at serine, threonine or tyrosine residues, (iv) initial mass accuracy tolerances = 10 ppm for precursor ions and 20 ppm for product ions, and (v) one missed cleavage allowed. In addition, the following criteria were used for protein identification: (i) ≥ 3 fragment ions per peptide, (ii) ≥ 7 fragment ions per protein, and (iii) ≥ 1 peptide per protein. Raw data and PLGS search results were imported into the Rosetta Elucidator software (build 3.3.0.1.SP3.19, Rosetta Biosoftware; Seattle, WA, USA). Elucidator performed retention time (RT) and mz/charge alignment, feature identification and extraction for all samples using the Rosetta PeakTeller algorithm. Dynamic background subtraction, smoothing in RT and m/z dimensions and isotopic regions creation for peak-matching across all runs were calculated using an RT correction of 4 min at the maximum. A single data file was randomly chosen as the master, and all other sample files were aligned to the master in form of a dynamic RT shift. This procedure allowed the improved identification of peptides and proteins in each sample by taking the available data of all samples into account. Features were filtered for high score and normalized based on total ion current (TIC). Only peptides detected in both replicates and in >80 % of samples were included in further analysis.

Protein abundance changes were determined using the MSstats package (Clough *et al*, 2012) based on linear mixed-effects models on the peptide intensities, following log_2_ transformation and exclusion of intensity values deviating more than three standard deviations from the mean of each group (<1% of total data). Proteins were identified by at least two peptides. The *p*-values were adjusted to control the false discovery rate (FDR) at a cut-off of 0.05 following the Benjamini Hochberg procedure. (Chang *et al*, 2012)

**Label-based selected reaction monitoring (SRM) mass spectrometry**

Abundance alterations of a panel of 43 candidate proteins implicated in the pathway analysis of the *Tsc1*^+/-^ mouse were measured using targeted SRM mass spectrometry on a Xevo TQ-S mass spectrometer (Waters Corporation) coupled online through a New Objective nanoESI emitter (7 cm length, 10-mm tip; New Objective) to a nanoAcquity UPLC system (Waters Corporation). The system was comprised of a C18 trapping column (180umx20mm, 5μm particle size) and a C18 BEH nano-column (75umx200mm, 1.7mm particle size). The separation buffers were (A) 0.1% formic acid and (B) 0.1% formic acid in acetonitrile. For separation of peptides, the following 48 min gradient was applied: 97/3% (A/B) to 60/40%B in 30 min; 60/40% to 15/85% in 2 min; 5 min at 15/85%; returning to the initial condition in 1 min. The flow rate was 0.3µL/min and the column temperature was 35°C.

SRM assays were developed following a general high-throughput strategy (Picotti *et al*, 2009). For method refinement, initially up to 12 unique peptides ranging from 6 to 20 amino acids in length, containing tryptic ends and no miscleavages were chosen for each of the selected proteins. All peptides containing amino acids prone to undergo modifications (e.g., Met, Trp, Asn and Gln), potential ragged ends, lysine/arginine followed by proline or bearing the NXT/NXS glycosylation motif were generally avoided and only selected when no other options were available (Lange *et al*, 2008). Peptides were checked by Protein BLAST (<http://blast.ncbi.nlm.nih.gov/Blast.cgi>) searches to ensure uniqueness. For method refinement, up to 12 transitions per peptide were tested in SRM mode. Transitions were calculated using Skyline version 1.2.0.3425 (MacLean *et al*, 2010) and corresponded to singly charged y-ions from doubly or triply charged precursors, in the range of 350-1250 Da. Transitions were selected based on software internal predictions, discovery proteomics data and spectral data available through the Human NIST spectral libraries (Farrah *et al*, 2011). Method refinement was performed on quality control samples. For the final SRM assays, the 2-3 peptides with the maximal intensities and highest spectral library similarity (dotp) were selected. A further development step, analyzing heavy-label spiked QC samples in scheduled SRM mode, was used to confirm identity via co-elution, extract the optimal fragment ions for SRM analysis, obtain accurate peptide retention times and optimize collision energy and cone voltage for the quantification run applying skyline software (MacCoss Lab Software; Seattle, WA, USA) (MacLean *et al*, 2010). Heavy labelled forms of these selected peptides (spiketides L) were chemically synthesized via SPOT synthesis (JPT Peptide Technologies GmBH, Berlin, Germany). The final transitions, collision energy and retention time windows used for each peptide can be requested from the corresponding author.

Quantitative SRM measurements comparing patients and controls were performed in scheduled SRM acquisition mode, using the optimized parameters defined during the assay refinement. For each target peptide a heavy isotope labelled internal standard (JPT Peptide Technologies GmbH) was spiked in the peptide mixture for accurate quantification and identification. All SRM functions had a 2 min window of the predicted retention time and scan times were 20 ms, which ensured a dwell time of over 5 ms per transition. For each peptide at least three transitions were monitored for the heavy and light version. Samples were run randomized and blocked (Oberg and Vitek, 2009) in triplicates and blanks and quality control peptide injections (yeast alcohol dehydrogenase) were performed alternating after every biological replicate. Resulting SRM data was analyzed using skyline and protein significance analysis was performed using SRMstats (Chang *et al*, 2012). In the first step, data pre-processing was performed by transforming all transition intensities into log_2_-values. Then a constant normalization was conducted based on reference transitions for all proteins, which equalized the median peak intensities of reference transitions from all proteins across all MS runs and adjusted the bias to both reference and endogenous signals. Protein level quantification and testing for differential abundance among patient and control groups were performed using the linear mixed-effects model implemented in SRMstats. The scope of validity of our conclusions was restricted to the specific biological replicates in the experiments. Each protein was tested for abundance differences between patients and healthy controls. The *p*-values were adjusted to control the FDR at a cut-off of 0.05 according to Benjamini and Hochberg (Chang *et al*, 2012).
