# Supplementary material for: A brain proteomic investigation of rapamycin effects in the Tsc1+/− mouse model
Source: Mol Autism. 2017 Aug 1;8:41. doi: 10.1186/s13229-017-0151-y (PMC5540199; doi:10.1186/s13229-017-0151-y)
Supplement: Supplementary file 3 — Full information for significantly changed proteins identified by label-based LC-SRM in the frontal cortex and hippocampus of Tsc1 +/− mice compared to wildtype mice. (DOCX 31 kb) [file 13229_2017_151_MOESM3_ESM.docx]

|  |  | **Frontal Cortex** | | | | | | |  | **Hippocampus** | | | | | | | |
| --- | --- | --- | --- | --- | --- | --- | --- | --- | --- | --- | --- | --- | --- | --- | --- | --- | --- |
|  |  | **SRM**  **(15 *Tsc1*^+/-^ / 15 wildtype)** | | |  | **MS^E^**  **(14 *Tsc1*^+/-^ / 15 wildtype)** | | |  | **SRM**  **(15 *Tsc1*^+/-^ / 15 wildtype)** | | |  | **MS^E^**  **(15 *Tcs1*^+/-^ / 15 wildtype)** | | |  |
|  |  |  | **Ratio** |  |  |  | **Ratio** |  |  |  | **Ratio** |  |  |  | **Ratio** |  |  |
|  |  |  |  |  |  |  |  |  |  |  |  |  |  |  |  |  |  |
|  |  |  |  |  |  |  |  |  |  |  |  |  |  |  |  |  |  |
| **Protein** |  |  |  | ***p*** | ***p**** |  |  | ***p*** | ***p**** |  |  | ***p*** | ***p**** |  |  | ***p*** | ***p**** |
| **Energy metabolism** |  |  |  |  |  |  |  |  |  |  |  |  |  |  |  |  |  |
| Aspartate aminotransferase | AATM |  | *n.s.* | | |  | *n.s.* | | |  | *n.s.* | | |  | *n.s.* | | |
| Ca2+ binding mitochondrial carrier | CMC1 |  | *n.s.* | | |  | *n.s.* | | |  | 1.31 | 1.6E-05 | 7.6E-05 |  | *n.s.* | | |
| Pyruvate kinase, mito. | KPYM |  | 1.08 | 8.4E-05 | 1.3E-03 |  | *n.s.* | | |  | *n.s.* | | |  | *1.03* | *0.025* | *0.083* |
| Hypoxanthine-ribonuclease | HPRT |  | *n.s.* | | |  | *n.d.* | | |  | 1.18 | 2.2E-04 | 7.2E-04 |  | *n.s.* | | |
| **Neurotransmitter metabolism/transport** | |  |  |  |  |  |  |  |  |  |  |  |  |  |  |  |  |
| Glutamate decarboxylase | DCE2 |  | 1.11 | 2.4E-02 | 1.5E-01 |  | *n.s.* | | |  | 1.24 | 1.3E-08 | 1.1E-07 |  | *n.s.* | | |
| GABA-aminotransferase | GABT |  | 1.06 | 5.0E-02 | 2.3E-01 |  | *1.02* | *0.024* | *0.106* |  | *n.s.* | | |  | *n.s.* | | |
| Proline dehydrogenase | PROD |  | *n.s.* | | |  | *n.d.* | | |  | 1.11 | 5.7E-02 | 9.4E-02 |  | *n.d.* | | |
| Catechol-O-methyltransferase | COMT |  | *n.s.* | | |  | *n.d.* | | |  | 1.16 | 2.9E-03 | 7.4E-03 |  | *n.d.* | | |
| Vesicular glutamate transporter 1 | VLGLU1 |  | *n.s.* | | |  | *n.d.* | | |  | 1.05 | 3.1E-02 | 6.1E-02 |  | *n.d.* | | |
| **Clathrin mediated exo-/endocytosis** |  |  |  |  |  |  |  |  |  |  |  |  |  |  |  |  |  |
| AP-2 complex subunit beta | AP2B1 |  | *n.s.* | | |  | *0.98* | *0..036* | *0.138* |  | 1.07 | 0.047 | 0.082 |  | *1.04* | *0.026* | *0.086* |
| Synaptojanin 1 | SNJ1 |  | *n.s.* | | |  | *n.s.* | | |  | *n.s.* | | |  | *n.s.* | | |
| Synapsin 1 | SYN1 |  | *n.s.* | | |  | *n.s.* | | |  | *n.s.* | | |  | 0.95 | 4.8E-05 | 9.5E-05 |
| Synaptotagmin 1 | SYT1 |  | *n.s.* | | |  | *n.s.* | | |  | *n.s.* | | |  | *n.s.* | | |
| **Long-term potentiation/signal transduction** | | | | |  |  |  |  |  |  |  |  |  |  |  |  |  |
| CamK2β | KCC2B |  | *n.s.* | | |  | *n.s.* | | |  | 1.08 | 4.8E-02 | 8.2E-02 |  | 0.94 | 5.0E-04 | 8.7E-04 |
| Calcineurin subunit B type 1 | CANB1 |  | *n.s.* | | |  | *n.s.* | | |  | 0.83 | 5.9E-09 | 6.3E-08 |  | *n.s.* | | |
| Neurochondrin | NCDN |  | *n.s.* | | |  | 0.91 | 2E-06 | 1E-04 |  | *n.s.* | | |  | 0.94 | 4.5E-03 | 7.0E-03 |
| Protein kinase C γ type | KPCG |  | *n.s.* | | |  | *n.s.* | | |  | 1.08 | 3.9E-02 | 7.3E-02 |  | *1.05* | *0.09* | *0.211* |
| PP2BB | PP2BB |  | *n.s.* | | |  | *n.s.* | | |  | *n.s.* | | |  | *n.s.* | | |
| Disks large homolog 4 (PSD95) | DLG4 |  | *n.s.* | | |  | *n.d.* | | |  | *n.s.* | | |  | *n.s.* | | |
| ERK1 | MK01 |  | *n.s.* | | |  | *n.s.*  *n.s.* | | |  | *n.s.* | | |  | *n.s.* | | |
| Astrocytic phosphoprotein PEA15 | PEA15 |  | *n.s.* | | |  |  |  |  |  | 1.23 | 8.7E-06 | 4.7E-05 |  | *n.d.* | | |
| **mTOR translation** |  |  |  |  |  |  |  |  |  |  |  |  |  |  |  |  |  |
| mTOR kinase | MTOR |  | *n.s.* | | |  | *n.d.* | | |  | 1.30 | 1.3E-03 | 3.5E-03 |  | *n.s.* | | |
| 60S ribosomal protein | RL8 |  | *n.s.* | | |  | *n.d.* | | |  | *n.s.* | | |  | *n.d.* | | |
| 40S ribosomal protein S3a | RS3A |  | 1.33 | 2.1E-04 | 1.8E-03 |  | *n.d.* | | |  | 1.28 | 1.7E-08 | 1.3E-07 |  | *n.d.* | | |
| **Oxidative stress** |  |  |  |  |  |  |  |  |  |  |  |  |  |  |  |  |  |
| Superoxide dismutase [Cu-Zn] | SODC |  | *n.s.* | | |  | *n.d.* | | |  | 0.90 | 7.8E-05 | 3.0E-04 |  | 0.96 | 1.9E-02 | 2.5E-02 |
| **Cell morphology/structural elements/synaptic plasticity** | | | | | |  |  |  |  |  |  |  |  |  |  |  |  |
| Sh3 & multiple ankyrin | SHAN3 |  | *n.s.* | | |  | *n.d.* | | |  | 1.23 | 1.0E-02 | 2.3E-02 |  | *n.d.* | | |
| Neural cell adhesion molecule 1 | NCAM1 |  | *n.s.* | | |  | *n.d.* | | |  | *n.s.* | | |  | *n.s.* | | |
| Neuromodulin | NEUM |  | *n.s.* | | |  | *n.s.* | | |  | *n.s.* | | |  | *n.s.* | | |
| Neurofilament light polypeptide | NFL |  | *n.s.* | | |  | 1.04 | 1.1E-03 | 1.6E-02 |  | *n.s.* | | |  | *n.s.* | | |
| Vesicle-fusing ATPase | NSF |  | *n.s.* | | |  | *n.s.* | | |  | 1.06 | 6.2E-03 | 1.5E-02 |  | 1.07 | 4.2E-09 | 1.2E-08 |
| Copine 6 | CPNE6 |  | 1.15 | 7.8E-02 | 2.3E-01 |  | *n.d.* | | |  | 1.26 | 5.9E-05 | 2.6E-04 |  | 1.10 | 6.6E-03 | 9.8E-03 |
| Actinin-1 | ACTN1 |  | *n.s.* | | |  | *n.s.* | | |  | 1.17 | 1.1E-02 | 2.4E-02 |  | *n.s.* | | |
| Actinin-2 | ACTN2 |  | *n.s.* | | |  | *n.d.* | | |  | 1.20 | 1.1E-03 | 3.3E-03 |  | *n.s.* | | |
| Cofilin-1 | COF1 |  | *n.s.* | | |  | *n.s.* | | |  | *n.s.* | | |  | *n.s.* | | |
| Profilin-1 | PROF1 |  | *n.s.* | | |  | *n.s.* | | |  | 1.46 | 1.9E-07 | 1.2E-06 |  | *n.s.* | | |
| Microtubule-associated protein 2 | MAP2 |  | *n.s.* | | |  | 1.04 | 6.1E-05 | 2.0E-03 |  | *1.04* | *7.5E-02* | *1.1E-01* |  | 1.05 | 4.2E-03 | 6.5E-03 |
| MARCKS | MARCS |  | 1.09 | 5.4E-08 | 2.3E-06 |  | *n.s.* | | |  | 0.87 | 4.2E-04 | 1.3E-03 |  | 0.94 | 0.012 | 0.052 |
| Transforming protein RhoA | RHOA |  | 1.09 | 2.1E-02 | 1.5E-01 |  | *n.s.* | | |  | *n.s.* | | |  | *n.s.* | | |
| Ras-related protein Rab-35 | RAB35 |  | *n.s.* | | |  | *n.s.* | | |  | 1.13 | 1.1E-04 | 4.0E-04 |  | *n.s.* | | |
| Transcript. activator protein Pur-α | PURA |  | 1.22 | 6.9E-02 | 2.3E-01 |  | 1.06 | 2.5E-03 | 2.9E-02 |  | excluded | | |  | *0.91* | *0.021* | *0.075* |
| **Oligodendrocyte-specific** |  |  |  |  |  |  |  |  |  |  |  |  |  |  |  |  |  |
| Myelin proteolipid protein | MYPR |  | 1.06 | 1.8E-04 | 1.8E-03 |  | 1.06 | 1.6E-05 | 7.7E-04 |  | 0.87 | <E-15 | <E-15 |  | 0.93 | 3.9E-08 | 1.1E-07 |
| Myelin basic protein | MBP |  | 1.11 | 9.0E-05 | 1.3E-03 |  | *n.s.* | | |  | 0.78 | <E-15 | <E-15 |  | 0.93 | 2.6E-05 | 5.8E-05 |
| Tetraspanin-2 | TSN2 |  | 1.03 | 8.4E-02 | 2.3E-01 |  | *n.s.* | | |  | 1.08 | 1.2E-02 | 2.4E-02 |  | *n.d.* | | |
|  |  |  |  |  |  |  |  |  |  |  |  |  |  |  |  |  |  |

**Supplementary Table S2: Full information for Table 1**
